# Evaluation of Behavior Change Communication Campaigns to Promote Modern Cookstove Purchase and Use in Lower Middle Income Countries

**DOI:** 10.3390/ijerph15010011

**Published:** 2017-12-22

**Authors:** William Douglas Evans, Michael Johnson, Kirstie Jagoe, Dana Charron, Bonnie N. Young, A. S. M. Mashiur Rahman, Daniel Omolloh, Julie Ipe

**Affiliations:** 1 Department of Prevention and Community Health, Milken Institute School of Public Health, The George Washington University, Washington, DC 20052, USA; 2 Berkeley Air Monitoring Group, Berkeley, CA 94704, USA; mjohnson@berkeleyair.com (M.J.); kajagoe@gmail.com (K.J.); dcharron@berkeleyair.com (D.C.); Bonnie.Young@colostate.edu (B.N.Y.); 3 Micro Industries Development Assistance and Services (MIDAS), Dhaka 1209, Bangladesh; mashiur@midas.org.bd; 4 Eco Consultancy, Nairobi, Kenya; daniel.omolloh4@gmail.com; 5 The Global Alliance for Clean Cookstoves, Washington, DC 20006, USA; jipe@cleancookstoves.org

**Keywords:** modern cookstoves, behavior change communication, social marketing, outcome evaluation, low and middle income countries, Bangladesh, Kenya, Nigeria, public health

## Abstract

Nearly three billion people worldwide burn solid fuels and kerosene in open fires and inefficient stoves to cook, light, and heat their homes. Cleaner-burning stoves reduce emissions and can have positive health, climate, and women’s empowerment benefits. This article reports on the protocol and baseline data from the evaluation of four behavior change communication (BCC) campaigns carried out in lower to middle income countries aimed at promoting the sale and use of cleaner-burning stoves. Interventions implemented in Bangladesh, Kenya, and Nigeria are using a range of BCC methods including mass media, digital media, outdoor advertising, and inter-personal communication. The mixed methods evaluation comprises three large-scale surveys: one pre-BCC and two follow-ups, along with smaller scale assessments of stove uptake and patterns of use. Baseline results revealed varying levels of awareness of previous promotions and positive attitudes and beliefs about modern (i.e., relatively clean-burning) cookstoves. Differences in cookstove preferences and behaviors by gender, socio-demographics, media use, and country/region were observed that may affect outcomes. Across all three countries, cost (lack of funds) a key perceived barrier to buying a cleaner-burning stove. Future multivariate analyses will examine potential dose-response effects of BCC on cookstove uptake and patterns of use. BCC campaigns have the potential to promote modern cookstoves at scale. More research on campaign effectiveness is needed, and on how to optimize messages and channels. This evaluation builds on a limited evidence base in the field.

## 1. Introduction

Globally an estimated 2.8 billion people rely on solid fuels such as wood, animal dung, crop waste, and charcoal, as well as kerosene for cooking, lighting and heating their homes [1]. Persistent exposure to household air pollution from solid fuels and kerosene accounted for nearly 2.6 million premature deaths as a result of pneumonia, cardiovascular disease and cancers [2]. Women and children are disproportionately affected due to greater time spent in or near the kitchen. The use of solid fuels and kerosene on rudimentary inefficient cookstoves also increases pressures on natural resources, contributing to environmental degradation, resource depletion, and climate change through CO_2_ and black carbon emissions, as well as other hazardous pollutants. Time spent cooking and the procurement of large amounts of fuel frequently takes the cook, mostly women, away from income generating activities or better education. Furthermore, the fuel costs are often much higher with traditional and inefficient stoves. On the other hand, clean cooking technology has the potential to make a substantial contribution to income and other economic activity [3]. Clean cooking options include improved biomass stoves, electric stoves, liquefied petroleum gas (LPG), and other cleaner-burning fuels, such as pellets or briquettes.

In the last sixty years, social marketing, behavior change communications (BCC), and related strategies to change behavior have taken a prominent place in addressing global health challenges [4]. Applying commercial marketing principles to social issues, social marketing and BCC interventions have been successfully used to encourage healthy behaviors including condom use, family planning, and water purification and sanitation [5]. These strategies have been shown to be widely effective in promoting behavior change across a range of subject matter, populations, and global settings [6,7]. 

Social marketing and BCC strategies have the potential to open up the market for modern cookstoves to the commercial sector over the long term through effective targeting of socially marketed product subsidies, through a process that has been termed the Total Market Approach (TMA) [8,9]. Following this approach, the Global Alliance for Clean Cookstoves and the United Nations Foundation have funded four projects to implement BCC campaigns to accelerate clean cooking markets by increasing awareness and adoption of clean cooking solutions, thus reducing health and environmental impacts of solid fuel and kerosene use.

The four projects are located in three countries:

(1) Bangladesh:
  - Community-based promotion of improved biomass cookstoves in peri-urban and rural areas (implemented by: Social Marketing Company and Purplewood Limited).
(2) Kenya:
  - Technology/brand neutral campaign to promote the broad themes of awareness and adoption of cleaner cooking and a targeted campaign aimed at increasing purchase and use of improved charcoal stoves (implementing organizations: Population Services Kenya and Practical Action).
  - Home makeover TV and radio show, Shamba Chef, featuring a range of fuels and technologies and promoting clean cooking and nutrition (implemented by: Mediae.org).
(3) Nigeria:
  - Mass media campaign supported with inter-personal communication (IPC) to improve uptake of LPG in urban and peri-urban areas of Abuja and Lagos State (implemented by: Africare and McCann Global Health).

These projects were funded by the United Kingdom Department for International Development (DFID) for a period of two years from June 2016–June 2018.

In order to evaluate outcomes of the four projects, and to assess the conceptual model, the investigators are conducting a quasi-experimental study at three time points across the two-year implementation period. The full evaluation, inclusive of baseline, midline, and endline data, will investigate five main research questions:

(1) Are the implemented BCC campaigns effective in prompting people to purchase clean and efficient cookstoves (hereafter called “modern”) and improving proper cookstove usage?
(2) How much of the observed changes in behavior can be attributed to the BCC campaigns implemented?
(3) Is there a dose-response relationship between higher exposure to cookstove messages and the outcomes of cookstove purchasing and improved proper stove usage?
(4) Which aspects of the BCC campaigns were more effective than others? How does this vary by gender, socioeconomic status, and other demographic variables?
(5) What are the impacts of the BCC interventions on relative progress towards health, environment, gender, and livelihood goals?

The goals of the current study were to (1) describe the protocol for a cross-site evaluation of four BCC campaigns to promote modern stove purchase and use, and (2) assess the baseline data from these campaigns as a preliminary step to modeling BCC outcomes over time. In the following, we report on the initial evaluation protocol, including methods, measurements, and analysis plans. These components are inclusive of the full study and the midline and endline stages yet to be implemented. Additionally, we report results from the baseline data collection, completed in 2017. Using only the available baseline data, we estimated preliminary logistic regression models to examine associations between three outcomes of interest (1) intention to buy a modern stove, (2) previous ownership of a modern stove, and (3) ownership of more than one stove type (stove stacking)—with measured demographic and other potential explanatory variables, such as attitudes, awareness, and media exposure. While these analyses were conducted purely as a preliminary step to understand the nature of our baseline data, they will provide a framework for future studies once midline and endline data become available. 

## 2. Methods

### 2.1. Study Design

The underlying evaluation framework is a quasi-experimental design that assumes levels of BCC exposure will vary by campaign, and by medium of exposure. Following previous studies, we assume there will be natural variation in exposure to cookstove campaign messages [10,11]. This is due to variable resources and efforts of local BCC implementation partners, expenditures of resources, and consumer access to media sources (e.g., mobile phones, mass media). As a function of higher and lower levels of exposure, we anticipate that cookstove utilization outcomes measured will vary. This variation will enable us to create a measure of campaign exposure, a cookstove-messaging dosage index (CMDI), similar to an approach developed previously by the investigators [12]. 

The current study reports on the overall evaluation protocol. We also report on data and preliminary multivariate models that examine associations of cookstove use and related outcomes with prior exposure to cookstove marketing efforts implemented by local sales organizations before the baseline data collection. Future research will calculate a dose-response curve based on measured exposure to cookstove BCC efforts and follow-up survey data [13].

### 2.2. Measures

Exposure data: We will use two types of data to measure exposure and develop the CMDI: (1) self-reported exposure to messages along with recall of messages, and program-related terminology (e.g., taglines); and (2) external data from multiple sources to measure potential exposure to cookstove messages at the community, online, and media market level. 

The evaluation employs multiple time-point rapid population-based surveys and in-depth interviews in a smaller sub-group in order to assess levels of self-reported exposure and recall of messages from different BCC media. Using baseline data we examined:

- The number of previous cookstove message channels due to commercial marketing of stoves (radio, text messages (SMS), community agents etc.) the respondent was exposed to and the frequency of exposure to specific promotion executions;
- Unprompted message recall from radio listeners and the effect of changes in message content over time;
- The role played by word-of-mouth, e.g., consumer-to-consumer recommendation; and
- The respondent’s status on the “consumer journey” (consideration, active evaluation, purchase, use).

To develop reliable estimates of prior commercial cookstove campaign exposure, self-reported campaign awareness data will be compared to objective measures of exposure, such as broadcast reach maps [14] and community implementation data [13,15]. At the midline and endline data collection time points, when BCC campaign data are available, media exposure data will be captured through available mass media monitoring sources in each country, as well as through local program implementation databases, which are provided directly by the BCC implementation partners.

Response data: The main outcomes of interest are changes in knowledge, attitudes, and practices related to household cooking and cookstove purchase and use. To demonstrate causality, the rapid population-based survey conducted at the project’s inception will establish baseline knowledge, perceptions, and practices related to the content of cookstove marketing efforts. Topics include, but will not be limited to, the detrimental impacts of traditional cooking methods, a range of solutions to avoid these detrimental impacts, and pathways to access and correctly use modern cookstoves. Information sources that influence the population at baseline have also been explored. 

“Rapid” population-based surveys and in-depth interviews will be used in addition to focus group discussions (FGD). The implementation of a range of evaluation tools will allow for the assessment of the magnitude and frequency of the responses to the BCC campaign as well as for exploring the meaning and understanding of these. Data collection tools are described below. Rapid surveys measured:

- Measures of aided and unaided awareness of campaign messages. Recognizing the overlap with exposure measurement, this will be done in conjunction with the self-reported recall of messages.
- Scales for subjective and descriptive social norms and related knowledge, attitudes, and beliefs [16].
- Assessments of message receptivity, including perceived credibility and emotional response.

While not reported here due to space constraints, FGDs will also be used among a representative sample of school and community members who participated in the school club demonstrations to assess how community readiness, interpersonal communication, and behaviors were affected by the club demonstrations. 

### 2.3. Data Collection

Population-based Rapid Surveys: The first approach involves population-based rapid surveys at multiple time points during the project, with approximately 800–1000 respondents per survey period per country. The baseline surveys were conducted during the formative research prior to implementation of the BCC, midline and endline or post-intervention surveys will also be conducted.

Respondents were randomly selected *within assigned catchment areas* to ensure that they had the potential to be exposed to the appropriate BCC campaigns targeting their areas at midline and endline. A screening process narrowed the respondent pool according to pre-defined socio-demographic characteristics to ensure focus on the target population and to control for likely confounding factors. 

The aim of the baseline in-person survey was to gain a measure and understanding of several factors:

- Socioeconomic and demographic characteristics of the selected population;
- Baseline knowledge, attitudes, and practices (KAP) related to cooking appliances and fuels;
- Perceived barriers to uptake and use of modern cookstoves;
- Exploration of gendered stereotypes and norms that exist in relation to stove purchase and use;
- Sources of current modern cookstove related knowledge; and
- Level of exposure to selected BCC channels i.e., TV, radio, billboards.

The final draft versions of the survey were piloted in populations with similar demographic characteristics to the targeted population in each country to ensure that they were effective, understandable and culturally appropriate tools. Data were collected in participants’ homes using the electronic data collection platform ODK (https://opendatakit.org/). 

The surveys began with a brief introduction with in-depth sociodemographic data, such as area of residence, age, sex, marital status, education, occupation, housing characteristics, and similar information for the primary income earner. Country-specific socioeconomic status was assessed by housing material for Bangladesh, the Living Standards Measure for Kenya (groupings ranged from 1 to 10), and a summary of household items and materials for Nigeria (scores ranged from 10 to 49).

Stove use and fuel patterns were assessed by the number and types of stoves used for certain tasks, primary stove type, simultaneous use of stoves, and likes and dislikes of different stove types. The amount spent on fuel for the stoves was also explored. Respondents reported on issues surrounding decision making for large household purchases, defined as equal to or greater than KSH3000 (Kenyan shillings) for Kenya, 1800 BDT (Bangladeshi taka) for Bangladesh, or N7000 (Nigerian naira) for Nigeria (These prices were set based on the average cost of the implementation project promoted stoves.) The next section queried respondents on sources of information, specifically frequency and amount of exposure to TV, radio, mobile phone, Internet, and social media, such as What’s App or Facebook. 

The final section covered the respondent’s previous exposure to marketing for clean cooking campaigns. This section assessed whether the respondent had seen or heard various local advertisements for clean cooking, and what their awareness, attitudes, and beliefs were surrounding modern stoves. Also, in Kenya and Bangladesh, visual aids of the promoted and other “modern” stoves (i.e., pictures of stoves to be promoted in the subsequent BCC campaigns) were shown to participants in order to test for recognition of previous marketing efforts and define the focus of the questions. 

### 2.4. Midline Data Collection Plans

The midline data collection is the next planned step in the evaluation. The rapid surveys will be repeated for each of the four implementing projects in late 2017–early 2018. These post implementation surveys will also include assessments of exposure to and awareness of the projects BCC campaigns. Households that have purchased one of the promoted stoves and/or fuels since BC project inception will be identified and the motivating factors leading to that purchased explored. 

### 2.5. Endline Data Collection Plans

In addition to the rapid surveys the following data collection methods will be conducted at endline for each implementing project. 

In-depth Interviews: Purchasers vs. Non-Purchasers (*N* = 150 for each BC project). In a second approach, in-depth interviews will be conducted at the late intervention stages. A sub-sample of 150, will be selected from the sample of follow-up rapid survey respondents. These sub-samples will be categorized by those who purchased a new stove since the start of the BCC campaign (*N* = 75), and a group of controls who did not yet purchase a new stove, matched to stove purchasers based on socio-demographic characteristics (*N* = 75). These study groups will have more intensive follow-up including comprehensive surveys to collect key data used for exploring determinants and barriers to purchasing a new stove. 

Intensive stove use monitoring: Purchasers with small comparison non-purchaser group (*N* = 80 for each BC project). The third approach to the evaluation will include a random selection of 50 respondents from each of the sub-sample of stove purchasers described above. In addition to the comprehensive surveys, this group of 50 stove purchasers will also have Stove Use Monitors (SUMs) placed on all stoves in their home for 4–5 months, which will enable tracking of actual use of each stove. Another 20 homes without the new stove, but which match the demographic and fuel use profiles of the purchasers, will be monitored with SUMs simultaneously to provide a baseline. Should homes in this baseline sub-group opt to purchase an improved stove during the SUMs monitoring, only the data from the pre-purchase period will be used in the baseline analysis. A sub-sample of the 50 purchaser homes will be randomly selected for cooking event observation and FGD at the end of the monitoring period.

### 2.6. Study Locations and Timeline

Table 1 below details the study location, populations and timing for each baseline BCC data collection. 

### 2.7. Participant Recruitment

Sample selection was designed to reflect the target audiences of each BCC implementation program, as summarized in Table 2.

For all locations, the main participant was the family member who organized the cooking/home keeping. The sample was designed to capture participants who also carried out most of the cooking for the household and were involved at least in some part in the decision-making for larger HH (Household) purchases. If the main participant was in no way involved in the decision-making for larger household purchases, the main decision-maker was also interviewed. If the main decision-maker was not available, the household was not eligible. If the main participant had a housekeeper and therefore organizes the cooking and household but does not cook more than three times per week, the housekeeper/maid was interviewed for certain sections of the survey. In Bangladesh the main participant was a married women and the secondary participant her husband.

Interviews were conducted at weekends, evenings, and early mornings (as well as during the day) to ensure the sample included both men and women who work outside the home. Households were selected from the selected areas using a standard approach to avoid any bias or convenience sampling. Screening surveys were used to determine eligibility. Verbal consent was requested from any willing and eligible participants. The study protocol was approved by the MaGil Institutional Research Board (2017 Protocol number BCC-001).

### 2.8. Statistical Analysis

This baseline analysis explored the association of sociodemographic characteristics, media exposure, and stove awareness and attitudes with respect to three key outcomes: (1) intention to buy a modern stove (or LPG stove in Nigeria), (2) having ever owned a modern stove (or LPG stove in Nigeria) as shown in the survey with visual aids, and (3) number of stove types owned that are in working order. Outcome 1 was defined as planning to buy a modern stove within the next month versus no plans at all, possibly planning to buy one but not within a month, or unsure. Respondents were filtered for analysis if they had been previously exposed to the advertisement of modern cookstoves for Kenya and Bangladesh, or if they did not currently own an LPG stove for Nigeria. Outcome 2 was defined as those who had ever owned a modern stove versus those who had not. Outcome 3 was defined as those who owned two or more stove types versus those who only owned one stove type. 

Descriptive analyses summarized frequencies, percentages, means and standard deviations (SD) for outcomes and explanatory variables. All potential explanatory variables were assessed in crude logistic regression with each outcome per country using odds ratios (OR) and 95% confidence intervals (CI). Variables with a crude association of *p* ≤ 0.10 with each outcome of interest were included in multivariable analyses. Full models included all potential covariates, and reduced models took out explanatory variables that were significantly associated with each other. Final models were selected based on their relative quality compared to the other model options using the minimum Akaike information criterion (AIC) [17]. Given the exploratory nature of this analysis, covariates with a *p*-value of 0.10 or less are described for their associations with the outcomes in the final models. All statistical analyses were performed in SAS 9.4 (SAS Institute Inc., Cary, NC, USA).

## 3. Results

### 3.1. Descriptive Summary

The sociodemographic characteristics of the sample are reported in Table 3. Final sample totals were 792 for Bangladesh, 981 for Kenya, and 890 for Nigeria. The sample unit was considered to be a household where between one and three people were interviewed. The Bangladesh sample consisted of households within a 15 km radius of an “upazila” center (a geographical region used for administrative or other purposes), which were medium to small towns, although the term “peri-urban” is not typically used in this region. The Kenya sample was half urban (50.6%), with 34.9% and 14.6% living in peri-urban and rural areas, respectively. The Nigeria sample was made up of 55.1% urban and 44.9% peri-urban households. For all three countries, the majority of the main cooks and key respondents were female, 99.4% for Bangladesh, 89.0% for Kenya, and 94.8% for Nigeria. Bangladesh had a higher percentage of males identified as the decision maker for large household purchases (63.8%), while Kenya and Nigeria were still predominately female. Approximately half of all respondents were 18–30 years old for all three countries, and the maximum age was 50. 68.8% of the participants in Kenya were married, 85.8% in Nigeria. As dictated by the inclusion criteria all respondents in Bangladesh were married. Educational status of the main cook (or primary income earner for Nigeria) were relatively similar for all three countries: over a third had no formal education, some primary, or completed primary, approximately half had some secondary or completed secondary, and 5.6%, 13.8%, and 9.9% had completed vocational school, some university, or higher for Bangladesh, Kenya, and Nigeria, respectively. Socioeconomic status in Bangladesh was split into two groups based on housing materials: 22.6% had semi-pucca or lower level pucca homes with walls made of burnt bricks, metal, or concrete; and 77.4% had semi-kuccha or semi-pucca homes with walls made of burnt bricks, thatch, bamboo, or metal. Kenya’s socioeconomic status was measured by the Living Standards Measure, which ranges from 1 to 10 (10 reflects the highest living standards in this sample), with 34.6% in levels 4–5, 38.8% in levels 6–7, and 26.6% in levels 8–10. Nigeria’s socioeconomic status was measured by 25 household items and materials, and scores reported as continuous measures ranging from 10 to 49 (49 reflects the highest status), with a sample mean of 25.8 (SD 9.2). The number of people living and eating meals in the home, excluding infants, varied between the countries with a mean of 6.2 (SD 2.2) in Bangladesh, 3.8 (SD 2.1) in Kenya, and 5.3 (SD 2.7) in Nigeria (Table 3).

### 3.2. Awareness, Attitudes, Beliefs, and Behaviors with Cookstoves

Table 4 summarizes respondents’ awareness, attitudes, and beliefs surrounding traditional and modern cookstoves and clean fuels. The percentage of respondents who said they would like to use specifically LPG more often was 61.8% in Bangladesh and 91.6% in Nigeria. In Kenya, 11.3% of participants who were asked a broader question, “what stove they would have if they could have any” cited LPG as their choice. When asked about perceived barriers of using LPG more in Bangladesh and Nigeria, or perceived barriers of buying a modern stove in Kenya, the most common barrier was that they were too expensive. In Bangladesh, 39.2% reported that it is a long way to travel to re-fill the cylinders and it is difficult to get to the purchase place, and 13.9% reported that the fuel is not always available at the store. In Kenya, 12.1% of respondents said they do not know where to purchase a modern stove. For Outcome 1, the question of intention to buy a modern stove, respondents were filtered to those who had seen the advertisements of modern stoves in Bangladesh (*N* = 250) and Kenya (*N* = 661), or those who did not already own an LPG stove in Nigeria (*N* = 656) (Table 4). (Note: In Bangladesh, the number who reported awareness were in fact aware of previously promoted improved mud/concrete stoves and not the stoves planned for promotion in the BCC campaign). Only 9.6%, 20.0%, and 11.4% of respondents said they planned to buy one within the next month in Bangladesh, Kenya, and Nigeria, respectively. When queried as to why they were not planning to buy one within the next month, the majority of respondents cited not having enough money for Kenya (87.3%) and Nigeria (84.5%), whereas the most common response in Bangladesh was waiting until the current stove breaks (47.7%).

### 3.3. Cookstove Use Behaviors

Stove use behaviors are reported in Table 5. For Outcome 2 of having ever owned a modern stove or LPG stove, only five (0.6%) respondents in Bangladesh reported ever owning a modern cookstove as shown in a visual aid, 36 in Kenya (3.7%), and 142 (15.9%) in Nigeria reported having ever owned an LPG stove. For Outcome 3, respondents reported owning two or more different stove types in Bangladesh (59.9%), Kenya (66.1%), and Nigeria (29.0%). The primary stove type for all respondents, regardless of owning only one stove type or more than one, was as follows: largely traditional wood-burning in Bangladesh (99.0%), traditional charcoal (28.3%), 3-stone or modified 3-stone fire (33.0%), kerosene (19.1%), or LPG (17.8%) in Kenya, and mostly kerosene (87.9%) in Nigeria (Table 5). 

### 3.4. Multivariable Regression Results

Table 6, Table 7 and Table 8 summarize results from the multivariable logistic regression models after adjusting for relevant explanatory variables. The table displays odds ratios (OR), *p*-values, and confidence intervals (CI) for all estimates. For the first key outcome of intention to buy a modern stove within the next month, three variables remained significantly associated with the outcome in Kenya after adjusting for all variables in the model. Respondents had greater odds of planning to buy a new stove in the next month if they strongly agreed that traditional stoves were bad for their health and family’s health (OR 1.57, 95% CI 1.03, 2.42), if they had more frequent exposure to the radio of 4–7 days per week (OR 1.63, 95% CI 0.93, 2.86), and if they had at least one day of exposure to the Internet in the past week (OR 1.49, 95% CI 0.94, 2.37). In Nigeria, respondents had greater odds of planning to buy an LPG stove within the next month if they had a positive attitude toward clean cooking by wanting to tell others about it (OR 2.66, 95% CI 1.56, 4.55), and if they were in the younger age group of 18–30 years (OR 1.55, 95% CI 0.91, 2.65) (Table 6). This outcome was skipped for Bangladesh due the majority of the population being unaware of the promoted stoves and so were not asked about their intention to purchase. 

The second key outcome was exploring factors associated with respondents having ever owned a modern stove or LPG stove (Table 7). In Kenya, socioeconomic status according to the Living Standards Measure was significantly associated among the highest category of 8–10 (OR 3.40, 95% CI 1.26, 9.16), and exposure to the radio in the past week among those who listened 1–3 days (OR 0.07, 95% CI 0.02, 1.00), after adjusting for other variables. In Nigeria, greater odds for having owned an LPG stove was seen among those with positive attitudes towards LPG stoves, such as ability to afford one (OR 1.86, 95% CI 1.14, 3.03) and sharing information with the community (OR 1.73, 95% CI 1.15, 2.61), as well as a higher socioeconomic status (OR 1.06, 95% CI 1.04, 1.08), and awareness that traditional stoves are bad for one’s health and the family’s health (OR 1.91, 95% CI 1.25, 2.94). Similar analysis was not possible in the Bangladesh sample as only five respondents (0.6%) said they had previously owned a modern stove. 

The third key outcome was whether a respondent owned two or more stove types, compared to owning only one stove type (Table 8). In Kenya, greater odds for owning two or more stove types was seen among those with higher socioeconomic status in categories 8–10 (OR 2.31, 95% CI 1.57, 3.39), being a female decision maker in the home (OR 2.21, 95% CI 1.46, 3.36), being married (OR 1.94, 95% CI 1.47, 2.57), and having exposure to the radio in the past week (OR 1.56, 95% CI 1.10, 2.23). In Nigeria, similar covariates explained multiple stove types owned, including being able to afford to cook with LPG (OR 1.58, 95% CI 1.06, 2.37), having a higher socioeconomic status (OR 1.03, 95% CI 1.01, 1.05), and being married (OR 2.48, 95% CI 1.49, 4.14). In Bangladesh, a mix of sociodemographic and media exposure variables were associated with owning two or more stove types in adjusted models. Greater odds for the outcome were observed among those with higher educational status (OR 1.82, 95% CI 1.31, 2.52), having a semi-pucca home (OR 1.98, 95% CI 1.34, 2.94), and living in Madaripur (OR 1.80, 95% CI 1.26, 2.58) or Jhakokathi (OR 7.86, 95% CI 5.13, 12.06) compared to Faridpur, watching some TV in the past week (OR 1.66, 95% CI 1.17, 2.35), and having used the Internet at least once in the past week (OR 2.66, 95% CI 1.42, 5.00).

## 4. Discussion

As noted earlier, the goal of this study was to (1) describe the protocol for a cross-site evaluation of four BCC campaigns to promote modern stove purchase and use, and (2) assess the baseline data from these campaigns as a preliminary step to modeling BCC outcomes over time. Three key outcomes of intentions to purchase, previous modern stove ownership, and ownership of multiple stove types were explored to identify associations with a variety of sociodemographic characteristics, stove and fuel use patterns, and exposure to the media and clean cooking marketing campaigns. We chose to examine these outcomes because they are central to the overall evaluation objectives, and baseline data were able to provide useful information that will inform future follow up analyses. This study provides an initial look into an ongoing program to increase the effective use of social marketing and BCC methodologies in the cookstoves sector, and should be considered as a first step in that long-term research agenda [18].

With respect to the three key outcomes examined in the descriptive analyses and regression models, we can draw several overall conclusions. First, based on the regression models, radio is an important media platform for information for Kenya as higher levels of reported radio use were associated with each of the outcomes. 

Second, in all three countries, not being able to afford LPG or another kind of modern stove is the most frequently reported barrier. While absolute affordability (i.e., in relation to household income) is not a barrier that BCC can overcome, the issue may be more nuanced [19]. Many individuals in all three countries who report not being able to afford a modern stove, at the same time own other discretionary consumer items, such as mobile phones, motor bikes, TV, and other items. There are clearly trade-offs being made by consumers as they are the targets of commercial and social marketing efforts, and decide what products to buy. BCC efforts need to prioritize cookstove purchases and understand how best to increase the salience and perceived value of cookstoves relative to other consumer options [20].

There is also relatively low awareness that traditional stoves are bad for one’s health and that of family in each of the three countries. This presents a tremendous opportunity for public education for the BCC campaigns [21]. While health as a message frame may or may not be the most persuasive [22], it is important information as part of a larger set of benefits to use in promoting modern cookstoves. In particular, efforts such as the Shamba Chef program, and radio advertising in Nigeria and Kenya, that highlights effects of cookstoves in the home and benefits of switching, may be able to integrate health messages without framing specifically on the topic of health.

Access to the Internet and social media are still relatively low in all three countries. Access is almost certain to grow in coming years, but at present they are not the most readily useable platforms for campaigns among these study populations. At the same time, TV appears to be powerful platform, as evidenced by Shamba Chef in particular, which is currently rated number one for its time slot on Sunday evenings. However, the distribution of TV watching per week varies widely by location, and thus only reaches some target audiences.

Additionally, there are a number of specific barriers by country. For example, in Bangladesh, access to LPG (refill cylinders, available in stores) seems to be a major barrier. In Kenya, not knowing where to buy a modern stove is a barrier. In these two countries, both classified by the World Bank as low income [23], few have actually ever owned a modern stove before, whereas in Nigeria, a low-middle income country [23], a higher percentage (16%) have owned such modern stoves.

Furthermore, there is clear evidence from the baseline that attitudes and beliefs towards modern stoves are associated with key outcomes of stove use intentions (Outcome 2) and use behavior (Outcome 3). This suggests that BCC may work to increase cookstove adoption and use through changes in positive attitudes and beliefs toward the product. This hypothesis should be investigated in the follow up midline and endline data collections.

Finally, the role of sociodemographic variables should be explored in future analyses, as they were associated with each of the key outcomes. Gender, socio-economic status, area of residence, and marital status were all associated with one or more outcomes. For example, being female and having higher socio-economic status was associated with a higher probability of “stove stacking” (multi-stove ownership). Such sociodemographic categorizations both provide hypotheses of consumer profiles to examine in future analyses with midline and endline data, as well as opportunities for audience segmentation in future consumer targeting to increase cookstove purchase and use [24].

This study was a first step in an ongoing evaluation of BCC for cookstove marketing. Future steps in this evaluation include midline assessments and endline assessments of BCC outcomes. As such, this study should be considered in the context of broader efforts to extend the evidence base in BCC.

The study has several limitations. First, the data were collected only at one time point and are cross-sectional in nature. No causality can be inferred from any of the significant statistical associations. As such, results are suggestive and provide potential avenues for investigation at midline and endline follow-ups. Second, we do not currently have qualitative data, which may help to interpret our results in the future and should be examined and compared to the survey results using mixed methods approaches in future studies [25]. Third, data are based solely on self-report, and in particular such data on exposure to cookstove promotions are subject to selective attention bias [26], and should be validated as feasible in future studies using independent data [27]. Validation analyses are planned for the midline and endline cookstove evaluation studies.

Potential future research studies include “heavy-up” (media delivery manipulation) experiments to examine dose-response effects of higher levels of cookstove promotion on the key outcomes examined in the current study [28]. Also, combinations of social marketing strategies including price subsidies and placement/distribution approaches (e.g., providing more local outlets for cookstoves) should be examined in future BCC evaluations [29].

## 5. Conclusions

This study describes the study protocol and provides an initial look at data from an ongoing multi-site evaluation of social marketing and BCC to promote modern cookstove purchase and use. As this sector is critical to improving public health and gender equity, and fighting climate change, in low and middle income countries, the current study provides valuable new information. Social marketing and BCC are new to the sector, and more research needs to be done to optimize the effectiveness of these proven behavior change strategies. 

In particular, because BCC is so new to the cookstoves sector, there is a need to identify potential relationships between marketing efforts and purchase and use behaviors, understand the nature of those associations, and design appropriate communication and marketing strategies. Findings presented here provide initial evidence that exposure to promotions may affect cookstove attitudes, beliefs, and potential use. The midline and endline data from this study will help to answer questions about the effects of exposure to promotions over time on cookstove outcomes.

## Figures and Tables

**Table 1:** Overview of behavior change communications (BCC) baselines.

Details	Kenya	Bangladesh	Nigeria
Location	Central and western Kenya	Dhaka and Barisal divisions in central and southern Bangladesh	Lagos State and Abuja City, Nigeria
Area	Urban, peri-urban and rural	Peri-urban/rural	Urban and peri-urban
Timing	January–February 2017	April–May 2017	May–June 2017

**Table 2:** Summary of recruitment criteria.

Key Restrictions	Kenya	Bangladesh	Nigeria
Age	23–50 (main participant)	20–35 (main participant) 25–40 (husband)	18–40 (main participant)
Socio-Economic Class (SEC)	Living Standards Measure (LSM) 4–10	Low to lower middle income	SEC C2-D
Mass Media	Watched TV in past week: rural areas	None	Listened to radio in past week in selected areas
Fuels	No restrictions	Purchases at least some Household (HH) cooking fuel and no or minimal use of liquified petroleum gas (LPG)	No or minimal use of LPG

**Table 3:** Sample descriptive characteristics. (Freq. denotes Frequency.)

Response	Bangladesh (*N* = 792)		Kenya (*N* = 981)		Nigeria (*N* = 890)	
	Freq.	Percent	Freq.	Percent	Freq.	Percent
Area of residence or region/district						
Urban	0	0	496	50.6	490	55.1
Peri-urban	792 ^1^	100.0	342	34.9	400	44.9
Rural	0	0	143	14.6	0	0
Sex of main cook for the household						
Male	5	0.6	108	11.0	46	5.2
Female	787	99.4	872	89.0	844	94.8
Sex of decision maker for large household purchases ^2^						
Male	287	36.2	118	12.0	54	6.1
Female	505	63.8	862	98.0	834	93.9
Age group						
18–30 years	423	53.4	568	57.9	404	45.4
31–40 years	369	46.6	252	25.7	486	54.6
41–50 years	0	0	161	16.4	0	0
Marital status						
Married	792 ^2^	100.0	675	68.8	764	85.8
Not married	0	0	306	31.2	126	14.1
Education	Main cook		Main cook		Primary income earner	
No formal education, some primary, completed primary	282	35.6	372	37.9	347	39.0
Some secondary, completed secondary	466	58.8	473	48.22	455	51.1
Vocational school, some university, completed university, post-graduate	44	5.6	136	13.8	88	9.9
Bangladesh SES: Housing materials ^3^						
Semi-pucca/lower level pucca	179	22.6	--	--	--	--
Semi-kuccha/semi-pucca	613	77.4	--	--	--	--
Kenya SES: Living Standards Measure score ^4^						
Levels 4–5	--	--	339	34.6	--	--
Levels 6–7	--	--	381	38.8	--	--
Levels 8–10	--	--	261	26.6	--	--
Nigeria SES: SEC total ^5^(Continuous measure)	--	--	--	--	Mean: 25.8	SD: 9.2
Total number of people who eat an evening meal at home, excluding infants(Continuous measure)	Mean: 6.2	SD: 2.2	Mean: 3.8	SD: 2.1	Mean: 5.3	SD: 2.7

^1^ SES denotes socioeconomimc status. All respondents in Bangladesh lived within a 15 km radius of an upazila center (a geographical regions used for administrative or other purposes) which were medium to small towns. ^2^ Marriage was an inclusion criteria for Bangladesh enrollment. ^3^ Bangladesh SES approximated by housing materials: “Semi-pucca/lower level pucca. Walls: burnt bricks, metal/asbestos sheets, concrete Roof: burnt bricks, metal/asbestos sheets, concrete” versus “Semi-kuccha/semi pucca, Walls: thatch, bamboo, metal/tin/asbestos sheet, burnt brick.” ^4^ Kenya SES score measured by Living Standards Measure, a widely used marketing research tool of 1–10 categories based on items of material wealth, housing components, and lifestyle. Only groups 4–10 were eligible for the survey. 10 reflects the highest living standards, and 1 is the lowest. ^5^ Nigeria SES score measured by socioeconomic category based on a summary of household items and housing components. Scores range from 10 to 49.

**Table 4:** Awareness, attitudes, beliefs, and social norms about cookstoves and fuels.

Response	Bangladesh (*N* = 792)		Kenya (*N* = 981)		Nigeria (*N* = 890)	
	Freq.	Percent	Freq.	Percent	Freq.	Percent
Would you like to use LPG more?(Kenya: What stove type would you have if you could have any? Those who said LPG)						
Yes	183	61.8	111	11.3	720	91.6
No	113	38.2	NA	NA	61	7.8
Most commonly reported barriers to: using LPG more (Nigeria, Bangladesh) buying a modern stove (Kenya)						
It is too expensive, I do not have the money	279	94.3	801	82.3	698	96.9
I do not know where to purchase it	0	0	118	12.1	2	0.3
It is a long way to travel to re-fill the cylinders, cannot get to purchase place	116	39.2	0	0	5	0.7
The fuel is not always available at my store, cannot find the fuel.	41	13.9	0	0	9	1.3
Do not know	2	0.7	52	5.3	0	0
OUTCOME 1: Are you planning on buying a modern cookstove in the next month? ^1^	(*N* = 250)		(*N* = 661)		(*N* = 656)	
Yes in the next month	24	9.6	132	20.0	75	11.4
No, Yes but not in next month, Do not know	226	90.4	529	80.0	581	88.6
Why are you not planning on getting one in the next month?	(*N* = 220)		(*N* = 498)		(*N* = 566)	
I do not have the money	24	10.9	435	87.3	478	84.5
I do not know where to get one	12	5.5	5	1.0	1	0.2
I will wait until my current stove breaks	105	47.7	24	4.8	5	0.9
Do not need/Satisfied with current stove(s)	0	0.0	19	3.8	0	0.0
Not familiar with these, didn’t know about these until recently, not interested	7	3.2	4	0.8	51	9.0
Other: Not a priority, not enough space, because of my children, have not planned or budgeted, landlord won’t allow it, afraid of fires, explosions, burns	1	0.5	7	1.4	27	4.8
Do not know	25	11.4	2	0.4	3	0.5
Refused	46	20.9	2	0.4	1	0.2

^1^ Filtered to those who had been exposed to the advertisement of modern cookstoves (Kenya, Bangladesh), and filtered to those who currently did not already own an LPG stove (Nigeria). Nigerians were specifically asked if they plan to buy an LPG stove.

**Table 5:** Cookstove use behaviors.

Response	Bangladesh (*N* = 792)		Kenya (*N* = 981)		Nigeria (*N* = 890)	
	Freq.	Percent	Freq.	Percent	Freq.	Percent
OUTCOME 2: Have you ever owned a modern cookstove?	Like the stoves shown in Visual Aid?		Like the stoves shown in Visual Aid?		LPG stove?	
Yes	5	0.6	36	3.7	142	15.9
No, do not know	787	99.4	937	96.3	748	84.0
OUTCOME 3: Number of stoves types owned that are in working order						
Only one stove type	318	40.2	333	33.9	631	71.0
Two or more stove types	474	59.9	648	66.1	258	29.0
Primary stove type						
Traditional charcoal (metal, ceramic, Jiko charcoal)	0	0	277	28.3	42	4.8
3-stone fire, modified 3-stone	0	0	323	33.0	49	5.5
Traditional wood (mud stove with 1–2 combustion chambers, ceramic)	784	99.0	5	0.5	0	0
Kerosene	0	0	187	19.1	777	87.9
LPG	0	0	174	17.8	0	0
Promoted modern stove (Jiko Ecozoom, Burn Jikokoa, modern biomass stove with chimney)	8	1.0	5	0.5	0	0
Other: Electric, unspecified	0	0	9	0.9	5	0.6

**Table 6:** Results from multivariable logistic regression. OUTCOME 1: Intention to buy a modern stove (or LPG stove in Nigeria) ^1^.

Response	Odds Ratio *	*p*-Value	Lower 95% CI	Upper 95% CI
Kenya (*N* = 659) ^2^				
Traditional cooking stoves are bad for my health and that of my family				
Strongly agree	1.57	0.04	1.03	2.42
Agree, disagree, strongly disagree, do not know	reference			
New cookstoves like these (Visual Aid A) use less fuel which saves you money and/or time.				
Strongly agree	1.31	0.20	0.87	2.00
Agree, disagree, strongly disagree, do not know	reference			
How many days did you watch TV in the past week?				
1 or more	1.58	0.12	0.88	2.81
None	reference			
How many days did you listen to the radio in the past week?				
1–3 days	0.79	0.21	0.33	1.88
4–7 days	1.63	0.01	0.93	2.86
None	reference			
How many days did you use the Internet in the past week?				
1 or more	1.49	0.09	0.94	2.37
None	reference			
Nigeria (*N* = 656) ^3^				
Traditional cooking stoves are bad for my health and that of my family				
Strongly agree	0.95	0.86	0.525	1.714
Agree, disagree, strongly disagree, do not know	reference			
LPG cookstoves are safe to use				
Strongly agree	1.41	0.260	0.775	2.554
Agree, disagree, strongly disagree, do not know	reference			
LPG cookstoves are a clean way to cook				
Strongly agree	1.43	0.193	0.834	2.457
Agree, disagree, strongly disagree, do not know	reference			
I would like to tell people in my community about the importance of cleaner, more efficient cooking				
Strongly agree	2.66	<0.001	1.559	4.549
Agree, disagree, strongly disagree, do not know	reference			
Education of the income earner				
Completed secondary or higher	1.40	0.27	0.774	2.519
Some secondary, primary, or less	reference			
Age				
18–30 years	1.55	0.11	0.911	2.649
31–40 years	reference			
How many days did you use the Internet in the past week?				
1 or more	1.53	0.15	0.859	2.706
None	reference			

* Odds ratios for each country’s final model are adjusted for all variables shown. ^1^ Too many missing responses (*N* = 542, 70%) from this question in the Bangladesh survey. ^2^ Respondents filtered to those who were exposed to clean cooking marketing. ^3^ Respondents filtered to those who currently did *not* own an LPG stove.

**Table 7:** Results from Multivariable Logistic Regression. OUTCOME 2: Kenya & Bangladesh: Have you ever owned a modern stove like these? (Visual Aid A). Nigeria: Have you ever owned an LPG stove?

Response	Odds Ratio *	*p*-Value	Lower 95% CI	Upper 95% CI
Kenya (*N* = 973)				
Living Standards Measure ^1^				
Categories 6, 7	1.23	0.31	0.46	3.29
Categories 8–10	3.40	0.005	1.26	9.16
Categories 4, 5	reference			
How many days did you listen to the radio in the past week?				
1–3 days	0.13	0.07	0.02	1.00
4–7 days	0.63	0.31	0.30	1.36
None	reference			
How many days did you use social media in the past week?				
1 or more	1.32	0.50	0.59	2.95
None	reference			
Nigeria (*N* = 829)				
Traditional cooking stoves are bad for my health and that of my family				
Strongly agree	1.91	0.003	1.248	2.937
Agree, disagree, strongly disagree, do not know	reference			
I can afford to cook with LPG				
Strongly agree	1.86	0.01	1.139	3.028
Agree, disagree, strongly disagree, do not know	reference			
I would like to tell people in my community about the importance of cleaner, more efficient cooking				
Strongly agree	1.73	0.01	1.146	2.611
Agree, disagree, strongly disagree, do not know	reference			
Socioeconomic score (continuous measure) ^2^	1.06	<0.001	1.037	1.083
Area of residence				
Urban	1.31	0.19	0.87	2.0
Peri-urban	reference			
How many days did you use the Internet in the past week?				
1 or more	0.84	0.45	0.542	1.312
None	reference			
Bangladesh ^3^				

* Odds ratios for each country’s final model are adjusted for all variables shown. ^1^ Kenya socioeconomic score is measured by Living Standards Measure, a widely used marketing research tool of 1–10 categories based on items of material wealth, housing components, and lifestyle. Only groups 4–10 were eligible for the survey. 10 reflects the highest living standard. ^2^ Nigeria SES score measured by socioeconomic category based on a summary of household items and housing components. Scores range from 10 to 49. ^3^ Only 5 (0.6%) of the sample in Bangladesh responded “yes” to this outcome.

**Table 8:** Results from Multivariable Logistic Regression. OUTCOME 3: Ownership of two or more stove types.

Response	Odds Ratio Estimate *	*p*-Value	Lower 95% Confidence Limit	Upper 95% Confidence Limit
Kenya (*N* = 980)				
LSM				
Categories 6, 7	1.83	0.21	1.319	2.535
Categories 8–10	2.31	0.002	1.574	3.394
Categories 4, 5	reference			
Sex of decision maker in home for large purchases				
Female	2.21	<0.001	1.46	3.358
Male	reference			
Marital status				
Married	1.94	<0.001	1.47	2.57
Not married	reference			
How many days did you watch TV in the past week?				
1 or more	0.98	0.910	0.693	1.389
None	reference			
How many days did you listen to the radio in the past week?				
1 or more	1.56	0.01	1.098	2.227
None	reference			
Nigeria (*N* = 829)				
I can afford to cook with LPG				
Strongly agree	1.58	0.03	1.06	2.37
Agree, disagree, strongly disagree, do not know	Reference			
SES (continuous measure)	1.03	0.002	1.01	1.05
Marital status				
Married	2.48	<0.001	1.49	4.14
Not married	reference			
Bangladesh				
Education of the income earner				
Some secondary or higher	1.82	0.0003	1.31	2.52
Completed primary or less	reference			
House type				
Semi pucca, lower-level pucca	1.98	0.0007	1.34	2.94
Semi pucca, semi kuccha	reference			
Area of residence (district)				
Madaripur	1.80	0.008	1.26	2.58
Jhakokathi	7.86	<0.0001	5.13	12.06
Faridpur	reference			
How many days did you watch TV in the past week?				
1 or more	1.66	0.005	1.17	2.35
None	reference			
How many days did you use the Internet in the past week?				
1 or more	2.66	0.002	1.42	5.00
None	reference			

* Odds ratios for each country’s final model were adjusted for all variables shown.
